# Operative Conservation of the Ovary

**Published:** 1915-11

**Authors:** Sigmund Goldberg

**Affiliations:** Buffalo Gynealogist to German Hospital, Consult Gyneaologist to German Hospital Dispensary


### BUFFALO MEDICAL JOURNAL

Yearly Volume 71 NOVEMBER, 1915 No. 4

### ORIGINAL ARTICLES

**The right is reserved to decline papers not dealing with practical medical and surgical subjects, and such as might offend or fail to interest readers. Contributors are solely responsible for opinions, methods of expression and revision of proof.**

### Operative Conservation of the Ovary

Address delivered before Maimonides Medical Club

By DR. SIGMUND GOLDBERG, of Buffalo

Gynealogist to German Hospital, Consult Gyneaologist to German Hospital Dispensary.

It may be stated as an axiom that the ovary is the fundament of womankind. On its anatomic and physiologic integrity depends the weal or woe of woman, and the propagation of the human race. The ovary, for its size, in comparison with other organs, is one of the most complex of the human body It is invested by peritoneum. Beneath this is the proper fibrous covering of the organ, the **Tunica Albuginea,** analogous to a similar structure in the testes. It encloses a peculiar soft fibrous tissue, **Stroma,** very richly supplied with blood vessels. The most important elements of the ovary from a pathologic standpoint, are the Grafian vesicle.

These vesicles contain the ova. Varying in number from ten to twenty, in size from a pin's head to a pea. These vesicles have thin, transparent walls, and are filled with a clear, colorless, albuminous fluid. During their early development, they are small and deeply seated in the substance of the ovary. As they enlarge, they approach the surface, and when mature, form small projections on the exterior of the ovary beneath the peritoneum.

In **Gray's Anatomy,** we find a statement which I will unqualifiedly contradict, because where he says, "The formation, development and maturation of the Grafian vesicles and ova continue uninterruptedly from infancy to the end of the fruitful period of woman's life,'' the words **fruitful periods** should be stricken out.

The mind of medical man has ever been concerned with the status of the ovary as a whole. Ancient history is almost barren upon the subject of ovarian diseases. That some of the functions of these organs were known to early anatomists there can be no doubt, for as early as 200 B. C. female animals were castrated, as mentioned by Aristotle, and in the second century A. D. Galen described the ovaries under the name of **Testes Muliebres.** To be sure, they were uninformed as to the effect on them of menstruation, they attributing this process, again according to Aristotle, to a superfluity in the blood Concerning the possibility of an internal secretion, ancient history is as much of a blank on internal secretions as of any other ductless gland.

In modern times, we realize more and more that the total ablation of the ovaries, whether in health or disease, is a matter of paramount importance, for we now know, that in addition to the well-known function of ovulation, of equal if not greater importance is the ovarian internal secretion.

This internal secretion is a chemic substance now called a **Hormone,** and it is being more and more accepted as a truism that menstruation is brought about by Hormone action, and particularly all the factors connected with it are under Hormone control. A large proportion of the disabilities associated with menstrual function are known to be Hormonically induced. It is asserted that the ovarian Hormone is secreted bv a **Corpora Lutea.** It is my belief that the ovarian Hormone exerts a most remarkable influence, not alone from an anatomico-pathological standpoint, but also influence secondary sex characters, especially with regard to its co-relation—a remarkable instance of which has been described by doctors Pearl and Surface of the **Main Agricultural Experiment Station.** A cow which had produced three calves and given an abundant flow of milk, suddenly ceased to produce the secretion from the mammary glands, the udder rapidly shrank to a small size, and the animal began to show the external characteristics of a bull. After a lapse of eight months, the assumption of the male characters in various regions of the body was complete and perfect. A necropsy showed as the only gross abnormality a simple cystic condition of the ovary. Histologically and cytologically, these organs differed from the normal cow's ovaries in only one essential respect, namely, that they had no **Corpora Lutea,** and I can fully subscribe to the statement in an editorial in the **American Medical Journal** concerning this experiment that no one can read the interesting report of so unusual a manifestation without suggestion of what the findings may mean in relation to human experience.

The discovery of the principle Hormone action by Starling and Bayliss in 1902 may fairly be declared to have been an epoch-making event, and in Starling's most recent statement **(Proceedings of Royal Society of Medicine, 1914)** in which he asserts that Hormones may either cause the increase or decrease of functions or alteration of nutrition or rate of growth. Of course, at the present time, we are still much in the dark concerning all the functions of the ductless glands. Our ignorance is colossal, but we know some facts concerning them, and in certain cases may apply such knowledge clinically. It is therefore self-evident that the removal of the ovary **in totalis** robs the woman of her greatest physical asset. It may be asserted as to Tuffiar and Maute in the **Presse Medicale,** Nov. 23, 1914, that the suppression of the ovarian internal secretion independently of its menstrual function disarranges the entire cycle of the ductless gland system, the effect of which may be seen in metabolical disturbances which may lead to chronic obesity and invalidism. It has, up to the present time, not been demonstrated that the ovarian secretion or ovulation begins only at the time of puberty, and ceases at the menopause, but as studies along these lines continue, it is becoming more and more evident that the internal ovarian Hormone is secreted during the entire life of the female, it being but a question of degree during the various periods of her existence. Otherwise, how are we to explain the numbers of cases of precocious menstruation, as, for instance, the one recorded by Thomas, in his work on **Disease of Women,** of a child, four years nine months of age, having menstruated regularly from the age of twenty-one months, and reports further that he personally has known of a case in which menstruation began at eight months and continued regularly. He also reports instances as being on record in which menstruation began at birth, or within a month afterwards, and one case of undoubted authenticity is recorded in which menstruation, beginning at two years, parturition at full term occurred when mother was only eight.

So far as the mental effect of the removal of the ovaries in their entirety is concerned, thus interfering with the harmonious cycle of Hormone secretions, Frankel comments on the surprising discovery that in thirty-one of his thirty-seven cases of juvenile mental disease or feeble-mindedness, the genital organs showed pronounced infantile conditions. He asserts that there can be no question that both genital and mental abnormalities are the consequences of one trouble, defective functioning on the part of the ovaries. **Dementia Precox,** Imbecility, and Epileptic Insanity generally affect young persons during the stage of development, and there is much to sustain the assumption that lack of the ovarian secretions is what is wrong, keeping the girl from developing normally in body and mind.

Alfred Gordon assumes that same position in regard to the castration of women, and after enumerating the various psychic manifestations asserts that operative procedures on the ovaries should be undertaken with a due feeling of great responsibility, and the tendency should be to preserve, as much as possible of any amount of normal tissue found in the ovaries.

This, then, seems to be the trend of the modern mind toward the preservation of the ovary in whole or in part, not alone for its function of ovulation, but because of its co-equal important internal secretion. Even at the present time, not all men are agreed concerning this subject of an internal Hormone. No less a light than Charles A. L. Reed, in his **Textbook of Gynecology,** 1914, makes the following statement. Page 738, in his article on the **Menopause,** "**Making a mystery where there is none,**" some have assumed that during the menstrual years the ovaries secrete a certain substance which determines the menstrual flux, and ministers to female health. Napier and Christopher Martin have held that this hypothetical substance, being lacking at the menopause, gives rise to some of the symptoms of the climacteric. But it should be remembered shoals of men, women and children live in health without active ovaries, or with none at all, yet have good health, and that the climacteric is not a pathologic process, or the menopause a symptom."

This is the same language used in his edition of 1901, but it is the only recent work on Gynecology to take so radical and unprogressive a stand upon this question. None of the works to which I have had access, with the exception of two, namely, Crossen's **Diseases of Women,** and the fifth volume of Johnson's **Operative Surgery** gives to ovarian conservation a separate article, and illustrations of the method of procedure. This surely indicates a trend in the right direction.

Women of the present day are not sufficiently impressed with the importance of **operative conservation of the ovary.** They have not yet graduated from their time-honored fallacy that the only function of the ovary is procreation.

It has been no uncommon experience during my professional life, for women to consult me for the purpose of having their ovaries removed as a means for the prevention of future pregnancies. And a number of times when about to operate upon other organs they have requested me to remove their ovaries, as they considered them more of an annoyance than a necessity.

In comparison with the above, what a value the human male places on the analogue of the ovary, the testicles. Propose their removal, **in totalis,** for almost any cause, and immediately there is a riot. This was beautifully exemplified in a patient of mine of about sixty years of age, ailing with consequences of prostatic hypertrophy. That was at a time when this condition was being treated by what was then known as the Whitehead operation, which consisted in the extirpation of the testicles, the underlying theory being that this would produce an atrophy of the prostate gland. This operation I proposed to my patient. This seemed so horrible a procedure to him that his son, a prominent attorney, requested that before going further, Dr. Park should be called in consultation. This was done, and the first thing Dr. Park recommended was the Whitehead operation. I told Dr. Park that as I had already proposed that operation, that he should reiterate the proposal. I shall never forget the scene when Dr. Park made the proposition in that suave manner so characteristic of him. The patient looked steadily into the eyes of Dr. Park, and for several moments stillness pervaded the room. When my patient did speak, he paraphrased these words of Iago in Shakespeare's Othello, "He who steals my purse steals trash. 'Tis something, 'tis nothing. 'Twas mine, 'tis his, and has been slave to thousands. But he that steals from me my testicles robs me of that which enrichens him not but makes me poor indeed."

The history of oophorectomy is of considerable interest. The kings of ancient Lydia are said to have had it performed upon women in order that they might be employed in places of eunuchs—the actual nature of the operation is somewhat doubtful, as in some instances in all probability only the clitoris was removed. Although from those details procurable it may be inferred that at least some genuine oophorectomies were performed.

During the seventeenth century a Hungarian sow-gelder is said to have removed the ovaries from his daughter as a punishment for her frequent lapses from virtue. In the eighteenth century the operation was seriously discussed by John Hunter of England and John Bell of Edinburgh, although, owing to the high mortality of all intra-abdominal operations these men lacked the courage of their convictions, and were unwilling themselves to undertake a hitherto untried procedure. But their teachings bore fruit, and the first prearranged and successful operation upon an ovary was performed in 1809 by Ephraim McDowel of Kentucky, who had been one of Bell's pupils, and soon thereafter it became an established procedure.

In 1823 James Blondell of London suggested the operation in a paper presented to the Royal Society of Medicine of London. In this he suggested that the removal of the healthy ovaries would in all probability cure dysmenorrhea, and the menorrhagia which accompanies inversion of the uterus where amputation is not practicable.

In 1872 Dr. Robert Battey of Rome, performed the operation for removal of the healthy ovaries which he termed **normal oophorectomy**—note the word ''normal''—for the premature production of the menopause. Battey, during the remainder of his life, operated frequently on this indication to abolish thereby a painful and nervous class of symptoms.

There followed, as a result of Battey's teaching, a wave of sacrificial ovarian surgery which now has passed, and a conservative tide having for its object the conservation of one ovary, or at least part of an ovary, is rising. The sudden and stormy onset of the menopause is prevented or otherwise removed, and the woman does not feel, as she calls it, so totally different from other women.

Schroder, according to A. Martin, was the first to attempt to remove only the diseased portion of an ovary, leaving the apparently healthy part. Martin adopted this method of practice in cases of adherent appendages in which the patency of the tube could be demonstrated, and concluded that the removal of the diseased portions of the ovary did not affect recovery from the operation; that women who had suffered such partial removal of the adnexa, were no more liable to an extension of the disease **to the healthy** portion of the **resected organ** than women whose ovaries were normal; and, finally, that in all these cases of excision, menstruation persisted and conception was possible.

The foundation of the principle of ovarian conservation is that the functions of ovulation and Hormone secretion are a necessary influence in the protection of good health, and the underlying object of conservation is the restoration of good health. It is now a well-known and established fact that if a portion of healthy ovarian stroma is left, it continues the functions of the ovarian system, even to the extent of secreting its normal hormones and ova capable of impregnation. This has been demonstrated in a large number of cases in the practice of others and in quite a number of my own, in corroboration of which I will relate the following cases, which seem to me the most interesting in my experience.

Mrs. Anna B. consulted me April 1, 1910. She had been married nine years, was sterile, and her marital life was becoming more and more unpleasant, her husband neglectful, and threatening to leave her. Upon examination I found the right ovary enlarged, the left ovary tender and a tenderness in the appendical region. She entered the German Hospital by my advice, and upon operation the right ovary was removed, one half of the left, and the appendix.

(My method of resecting the ovary is somewhat different from the method I have seen used by others, and described in text-books aforementioned. I object to elliptical incision, because the ova-sacs gradually approach the surface, as we learned in our review of the anatomy of the ovary, and in making such elliptical incision we are of necessity incising ovary from the surface, toward the center and a number of degenerative cysts, especially in cases of cystic degeneration of the ovary, are sure to be missed. And this is the underlying cause of the reported five per cent of recurrences requiring secondary operation. No case of necessary secondary operation has occurred in my experience, because I cut the ovary horizontally, not ellipitically).

And now to return to the case. The patient made an uneventful operative recovery, and I attended her in labor, assisted by Dr. Mansfield G. Levy, on January 25, 1912, during the delivery of a nine pound male child.

In January, 1908, Mrs. K., consulted me, with following anannesis—nearly two years before she had been delivered of a still birth under the following circumstances. Her physician had called upon her, and telling her that she had plenty of time, left her, saying that he would return in a couple of hours. Shortly after his departure, and during his absence the child was born. I am uncertain whether it was directly a still birth, or whether the infant died immediately after birth. From her story I gathered that there was considerable hemorrhage, which was excessive until about the sixth day, when she was curreted by her physician, and all hemorrhage ceased. From that day there had been complete ammenorea, up to the day of her consulting me, notwithstanding constant medication and treatment. She remained under my care for nearly one year without my being able to relieve the ammenorea. One night I received a message that she was in great pain, and urgent for my immediate attention. Dr. Jesse Levy saw her for me during that night, and reported to me next morning that she was in all probability having an attack of appendicitis. I later confirmed this diagnosis, and directed her removal to the German Hospital. I made a median incision, assisted in this operation by Dr. Jesse Levy, instead of the usual right rectus, because here was an opportunity seldom vouchsafed the surgeon, namely, to witness **in vivo** ovaries functionally dead. After the removal of the appendix, the ovaries were found slightly smaller and considerably paler than normal. Bier's hyperemia treatment was then coming into vogue for various and diverse ailments, and it occurred to me that if I could produce an increased supply of blood to the ovaries, I might perform a unique act of surgical therapeutics. Accordingly, I massaged each ovary for several moments until they appeared to me redder in color and fuller to the tactile sense, softer in consistency. Before leaving the hospital, Mrs. K, had a normal menstruation, menstruating regularly for some months, when they ceased again for nine months, at the end of which time, assisted by Dr. Jesse Levy, I delivered her of a male child. She has had a number of children since.

One more case which I consider interesting and worthy of mention is as follows: Miss W. entered the German Hospital immediately after its opening for occupancy. Dr. Hartwig performed on her an appendectomy. She made an uneventful recovery. About three months later she consulted me for pain in both ovarian regions. Upon examination I found both ovarian regions full and tender. On my advice she re-entered the German Hospital, and upon operating, I did what was being universally done at that time: removed, as I believed, the ovaries **in totalis.** To my surprise, she continued to menstruate, and when she consulted me about a year later concerning the advisability of marriage, I told her that I thought her marital state would be devoid of motherhood, and that she should inform her prospective husband, for the sake of her future ease of mind, of the facts I had given her. She bluntly told me that she would marry, and that her husband would have to take his chances. Exactly one year after her marriage I delivered her of a beautiful specimen of female babyhood.

This, of course, brings up the question. Did I unintentionally leave behind some part of ovarian tissue? I feel positive that I did not. Some of these cases are explained by the supposition that there must have been a supernumerary ovary. If so, I knew nothing of it. It is believed, though, that even the ovarian ligament may contain some true ovarian stroma, and this may, in some cases, be the explanation.

Judging from my personal experience, and from what I have heard, read and seen in the work of others, I am unalterably convinced that the complete and absolute destruction of all normal ovarian tissue is one of the rarest of pathological phenomena. In almost every case, with rare exceptions, some part of some ovary may be conserved for the benefit of the woman. And from a social standpoint, the removal of ovaries **in totalis** is only too often the cause of domestic infelicity, and it has often been taken advantage of by men as a reason for breaking off engagements, and for the violation of the marriage vows, and the abandonment of wife and children.

In conclusion, I would formulate for your kindly consideration and friendly criticism the following propositions: The ovary, being an organ of such vital importance to the female economy, its operative conservation should ever be uppermost in the mind of the surgeon, because of its three-fold function: the causing of menstruation, the secreting of ova and hormones; second, because there is no condition, mechanical or pathological, except extremely rare, in which the ovary is so thoroughly incapacitated from its normal functioning, but that some part of it may be conserved in the interests of the patient; third, the percentage of recurrences after conservative operation will be greatly reduced, when straight horizontal incision is used instead of the more generally used elliptical; fourth, removal of ovaries does not produce deleterious effects because there is no longer a secretion of the ovarian hormone **per se**, but because its absence destroys that normal hormone cycle of all ductless glands.

508 Eagle Street.

---

**Achalasia of the Cardia.** A. F. Hertz (Quar. Jour. Med., Vol. VIII, No. 32), discusses cardiospasm using a new word achalasia, coined for him by Sir Cooper Perry and signifying an absence of relaxation. As a result of his studies, largely with the X-ray, the author believes there is a definite sphincter at the cardia which is in a state of contraction except when reached by food. Occasionally the sphincter fails to relax but the absence of true cardiospasm is shown by the easy passage and withdrawal of a mercury tube. Such a condition is functional, in some cases due to imperfect vagus control.

Diagnosis is best made by means of the X-ray, following a meal of gruel containing barium or bismuth.

Treatment is quite satisfactory by the passage of a mercury tube (20-24 cal.) before meals. In long standing cases with sac formation the swallowing of a silk thread may serve to direct the dilator.

Waterbrash, the author, explains as a certain form of achalasia with accumulation of saliva in the lower esophagus.

---

**Relation of Gas Bacillus to Diarrhoea.** P. H. Sylvester and F. H. Hibben, Boston, Arch. of Paed., June. 477 plants were made from 172 cases in infants, 369 plants and 100 cases being positive, 108 plants and 72 cases negative. Infectious diarrhoea: positive 38, negative 9; with 4 positive cases in mixed infection. Fat intolerance: positive 29, negative 3. Included in the general series are 30 breast fed infants under 2 weeks of age, all negative. They hold the gas bacillus to be pathogenic and not a normal inhabitant of the bowel. Fat-free, unpasteurized, lactic-acid milk is considered the best treatment.

---

**Relation of Syphilis to Sydenham's Chorea.** Henry Koplik of N. Y., Arch. of Paed., Aug., from negative results of Wassermann test and of diagnostic use of Salvarsan, concludes that such a relation does not exist—unless accidentally—and that salvarsan is not indicated.

